# Outcome of Buried Versus Exposed Kirchner Wires in Terms of Infection in Fractures of Phalanges and Metacarpal Bones of Hand

**DOI:** 10.7759/cureus.22515

**Published:** 2022-02-23

**Authors:** Husnain Khan, Ali Adil, Nur Ul Ain, Bilal A Qureshi, Umer F Chishti, Tayyab S Malik

**Affiliations:** 1 Plastic and Reconstructive Surgery, Holy Family Hospital, Rawalpindi, PAK

**Keywords:** kirchner wife, hand surgery, fracture fixation, phalanges, metacarpal, hand fracture, exposed k-wires, buried k-wires, pin site infection, k-wires

## Abstract

Introduction and objective

The fracture of hand bones is very common among manual hand workers and a fractured hand imparts a great effect on a person’s productivity both socioeconomically and from a body image point of view. The most common method of hand fractures fixation is with the help of Kirschner wires. Kirchner wires can be inserted in exposed or in buried manner. There are a few studies that provide a comparative analysis of rate of infection between these two techniques. This study aimed to assess the rate of infection in buried versus exposed Kirschner (K)-wires for hand fractures.

Material and method

The study was designed as a randomized controlled trial with consecutive non-random sampling. It was conducted in the Department of Plastic Surgery, Holy Family Hospital, Rawalpindi, Pakistan, and lasted from June to December 2019. Blinding was not possible as both the operating surgeon and patient were aware of the procedure being done; however, the assessor was blinded and was not aware which group got which treatment. Total 122 patients with fractures of metacarpals and phalanges of hand were included in the study and were divided into two groups with 61 patients in each. Group A was treated with buried K-wires and group B with exposed K-wires. The patients were followed for one month for the outcomes in terms of infection in the patients.

Results

Group A had 24 females (39.3%) and 37 males (60.7%). Group B had 16 females (26.2%) and 45 males (73.8%). In group A, nine (14.8%) patients had ages between 10 and 20 years, 18 (29.5%) patients between 21 and 30 years, 14 (23.0%) patients between 31 and 40 years, 11 (18.0%) patients between 41 and 50 years, and nine (14.8%) were between 51 and 60 years. The mean duration of surgery was 35.16 minutes for group A and 27.30 minutes for group B. Based on modified Oppenheim scoring system for pin site infection, out of 61 patients, seven (11.5%) with buried K-wires while 14 (23%) with exposed K-wires developed pin site infection.

Conclusion

Rate of infection is low in buried K-wires as compared to exposed K-wires though not statistically significant (p>0.05) for the fractures of metacarpals and phalangeal fractures of hand.

## Introduction

The common fractures of the upper extremity are phalanges and metacarpals fractures which account for 10% of all the fractures [1]. Among these, metacarpal fractures account for 36% of hand and wrist fractures [2]. These injuries halt one from the work and daily activities and can be complicated by stiffness and weakness. Multiple factors influence the indications of operative intervention, including stability, location, geometry, configuration, and associated injuries, but surgical intervention has been chosen when the benefits of surgery outweigh the outcome of non-operative management [3]. The aim of treating a fracture is to reduce and stabilize the fracture, maintain the reduction and start early rehabilitation to regain the lost function [4]. There are two methods of fracture reduction. The first method is open reduction which requires exposure of the fracture site and stripping of the periosteum. Stripping the periosteum causes scaring the fracture site and can reduce motion so when open reduction is planned, the fixation method should be stable enough to allow for early motion [5]. Another method is closed reduction in which the fracture is reduced with manipulation across the fracture and then it can be fixed internally with the help of Kirchner wires or nails and externally with the help of casts and splints. In this type of fracture management, hematoma formation at the fracture site is the main stimulus for bone healing. This hematoma causes inflammation at the fracture site, where osteoblasts and fibroblasts proliferate, resulting in the formation of a primary callus, which then gives rise to the formation of a hard callus. That hard callus formation ultimately enters the remodeling phase in which excess callus is reduced and bone strength is gradually increased with passage of time [6,7]. There are many methods of rigid fracture fixation including mini plates, compression plates, lag screws, Kirchner wires (exposed or buried) or cerclage wires, etc. depending on the type of fracture [8]. All of these methods have their merits and demerits, and preference of one technique over the other is subject to many factors like extent of soft tissue damage, open or closed fracture, time since injury, age of the patient, availability of the equipment, and surgeon’s preference [9].

Kirchner wire (K-wire) fixation is one of the commonly used fixation techniques for metacarpal and phalangeal fractures. These stainless steel or titanium wires are easily available and are cost-effective, especially in emergency settings. The K-wires provide reliable fixation and are simple yet very versatile, i.e., they can be used in various configurations across the fractures [10].

The fractures where rotation of the fractures segments is anticipated, K-wires can be inserted in cross or bi-planner fashion, e.g., (in phalangeal fractures) in axial fashion where rotation of the fracture segments is relatively less like in carpal and metacarpal fractures. When there is excessive soft tissue involvement, fractures across the joints, doubtful viability of the distal digital segment(s), gross contamination, and more comminution, the K-wires can be fabricated into external fixators in different configurations [11,12].

Although careful insertion of K-wire reduces the pin tract infection, it is a common complication of fracture fixation with this method. It is usually treated with local and systemic antibiotics, local wound care, debridement, and removal of K-wire. If left untreated it can lead to serious complications like osteomyelitis, early physeal fusion, flexor sheath infection, septic arthritis, and toxic shock syndrome [13,14]. The risk of K-wire infection increases with an increase in the time of K-wire left in situ [15].

The K-wires can be inserted with one end of the wire exposed or buried under the skin via a keyhole incision which is closed with sutures. Ahmad et al. in his study found 13 out of 66 (19.6%) wires in percutaneous K-wire group have developed infection in comparison to only two out of 88 (2.2%) wires in buried K-wire group. The difference was significant statistically (p <0.001) [16]. In another similar study, Rafique et al. found that the rate of infection was significantly low (4.4%) in buried K-wires as compared to exposed K-wires (18.2%) [17]. In one of the recent retrospective studies, Ridley et al. observed similar findings for the rate of infection in exposed versus buried K-wires (17.6% of exposed K-wires versus 8.7% of buried K-wires) [18].

In another recent study by Matthew et al., 53% of plastic surgeons and 41% of orthopedic surgeons preferred to leave the K-wire exposed after managing the fractures of hand bones. The surgeons preferred exposed K-wires based on ease of removal of the wires at follow-up and the surgeons who preferred buried K-wires expressed a perception of decreased infection rated in the buried K-wires group patients [19].

In a multicentric retrospective, cohort study in two tertiary care centers in Puducherry, it was shown that the infection rate was significantly lower in patients with buried K-wires (19.8%) compared to those with exposed K-wires (27.9%) for both open and closed types of fractures [20].

K-wire fixation is one of the common methods of managing metacarpal and phalangeal fractures of the hand. Although various international studies have proved the role of infection is low in buried K-wire. Our goal was to assess the outcome of buried and exposed K-wires in patients presenting to our hospital so that guidelines can be delineated to adopt a better procedure that will benefit patients in early recovery and rehabilitation by reducing the infection rate.

## Materials and methods

The study design was randomized controlled trial, and consecutive non-random sampling was used. Blinding was not possible as both the operating surgeon and patient were aware of the procedure being done; however, the assessor was blinded and was not aware which group got which treatment. The study duration lasted for six months, from June 2019 to December 2019. The study was conducted in the Department of Plastic Surgery, Holy Family Hospital, Rawalpindi, Pakistan. Total 122 patients with fractures of metacarpals and phalanges of hand were included in the study. The patients were divided into two groups with 61 patients in each. Group A was treated with buried K-wires and group B with exposed K-wires. The patients were followed for one month for the outcomes in terms of infection in the patients.

The sample size was calculated from WHO calculator with a 5% level of significance, 80% power of study, ratio (B:A) of sample size was 1, proportion of infection in group A (buried K-wire) 4.4%, and in group B (exposed K-wires) 18.2%. The required sample size was 122 (61 in each group). The sampling technique used was consecutive non-random sampling.

We included patients belonging to the age group of 14-60 years, of either gender, presenting with acute hand trauma and isolated fractures of metacarpals and phalanges of hand. Both open and closed fractures were included in the study. Patients having contaminated wounds (requiring serial debridement and associated with the high rate of infection), polytrauma, unstable patients already infected metacarpal and phalangeal fractures, and patients presenting 24 hours after trauma were excluded from the study.

Data collection procedure

**Figure 1:**
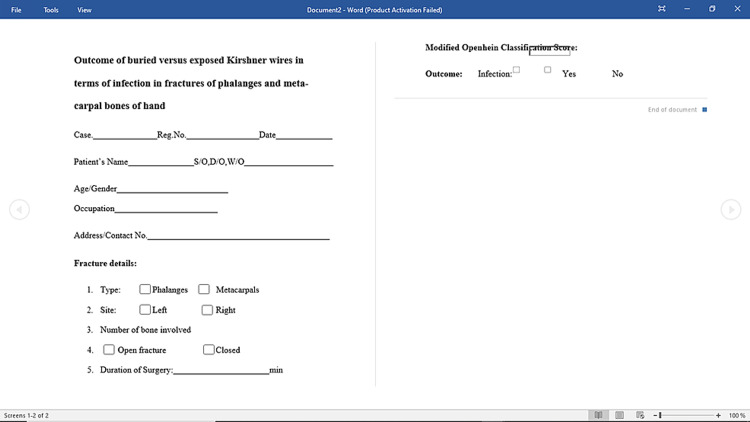
Structured questionnaire proforma.

After approval from the ethical committee and taking the informed consent, 122 patients fulfilling the inclusion and exclusion criteria were enrolled from the plastic surgery department. Demographic data including name, age, gender, address, phone number, registration number, and date of admission were noted. A clinical examination was performed. Subjects were assigned to one of the two treatments (exposed K-wires and buried K-wires) through a consecutive non-random sampling. We used coin flip to allocate patients to either group A or group B. The patients were divided into two groups, Group A and B. Group A underwent buried K-wire procedure and group B underwent exposed K-wire procedure. Same antiseptic measures, i.e., peri-operative antibiotics, pre-operative scrub, and pre-operative normal saline irrigation were administered to the patients peri-operatively and all the procedures were carried out by the same team. The patients were discharged after surgery and called for follow-up after two weeks for removal of stitches and after one month to measure the outcome of surgery by modified Oppenheim classification score (Table 1). All the information was entered in a structured questionnaire (Figure 1).

**Table 1:** Modified Oppenheim classification.

Grades	Clinical findings	Treatment
1	Slight discharge and redness around pin	Local pin and wound care
2	Redness and tenderness in soft tissues with or without discharge of pus	Local pin and wound care + oral antibiotics
3	As for grade 2 but with failure to improve with local care and oral antibiotics	Infected pin removed + oral antibiotics
4	Severe soft tissue involvement affecting more than one pin	Infected pin removed + oral antibiotics
5	As for grade 4 but also with bone involvement visible on x-ray	Pins removed + curettage of bone
6	A sequestrum has formed within the bone and persistent sinus has developed	Further surgery required to eradicate the problem

Data analysis procedure

The data were entered and analyzed by SPSS, version 22.0 (Armonk, NY: IBM Corp.). Quantitative variables like age and duration of surgery were presented as mean and standard deviation, and qualitative variables like gender and outcome in terms of infection for both procedures were presented as frequency and percentage. Chi-square test was applied to compare frequency of infection between the two groups. Data were stratified for age, gender, and type of fracture (phalanges/metacarpal), and post-stratification chi-square test was used to assess the outcome in both procedures with p <0.05 as statistically significant.

## Results

Total 122 patients fulfilling the inclusion criteria were included in the study after taking informed consent. The patients were divided into two groups. Group A was treated with buried K-wires and group B with exposed K-wires. Both groups contained 61 patients in each. Group A had 24 females (39.3%) and 37 males (60.7%). Group B had 16 females (26.2%) and 45 males (73.8%) (Table 2).

**Table 2:** Gender distribution of the subjects.

	Group A		Group B	
Gender	Frequency	Percent	Frequency	Percentage
Female	16	26.2	24	39.3
Male	45	73.8	37	60.7
Total	61	100.0	61	100.0

In group A, 10 (16.4%) patients had age between 10 and 20 years, 16 (26.2%) patients between 21 and 30 years, 14 (23.0%) patients between 31 and 40 years, 11 (18.0%) patients between 41 and 50 years, and 10 (16.4%) patients were between 51 and 60 years. In group B, nine (14.8%) patients had age between 10 and 20 years, 18 (29.5%) between 21 and 30 years, 14 (23.0%) between 31 and 40 years, 11 (18.0%) between 41 and 50 years, and nine (14.8%) were between 51 and 60 years of age (Table 3). 

**Table 3:** Age distribution of the subjects.

	Group A		Group B	
Age (years)	Frequency	Percent	Frequency	Percentage
10-20	10	16.4	9	14.8
21-30	16	26.2	18	29.5
31-40	14	23.0	14	23.0
41-50	11	18.0	11	18.0
51-60	10	16.4	9	14.8
Total	61	100.0	61	100.0

The mean duration of surgery was 35.16 minutes for group A and 27.30 minutes for group B (Table 4). In group A, 40 (65.6%) patients had closed while 21 (34.4%) patients had open fractures. In group B, 32 (52.5%) patients had closed while 29 (47.5%) patients had open fractures (Table 5).

**Table 4:** Duration of surgery for each group of subjects.

Duration of surgery	Minimum (minutes)	Maximum (minutes)	Mean (minutes)
Group A	15	120	35.16
Group B	15	60	27.30

**Table 5:** Incidence of closed versus open fractures in the subjects.

	Group A		Group B	
	Frequency	Percent	Frequency	Percentage
Closed	40	65.6	32	52.5
Open	21	34.4	29	47.5
Total	61	100.0	61	100.0

Based on modified Oppenheim scoring system for pin site infection, group A with buried K-wires had seven (11.5%) patients with infection while 54 (88.5%) patients had no infection. In group B, 14 (23%) patients had infection while 47 (77%) patients had no infection (Table 6).

**Table 6:** Incidence of pin site infection in each group.

	Group A		Group B	
Infection	Frequency	Percent	Frequency	Percentage
No	54	88.5	47	77.0
Yes	7	11.5	14	23.0
Total	61	100.0	61	100.0

By comparing the rate of infection in the two groups, we concluded that although the infection rate was low with buried K-wire group, but when analyzed statistically, this was not significant (p>0.05), so we accepted the null hypothesis (Table 7).

**Table 7:** Comparison of the infection in the study groups. Value of chi-square test was 2.82, degree of freedom was 1, and probability was 0.093.

Groups	Infection		Total	p-Value
	Yes	No		
A	7 (11.5%)	54 (88.5%)	61 (50%)	0.093
B	14 (23%)	47 (77%)	61 (50%)	0.093
Total	21 (17.2%)	101 (82.7%)	122 (100%)	

## Discussion

K-wire fixation is a common yet versatile method of internal fixation of fractures, especially in hand and wrist. It is also very commonly used for arthrodesis in the hand joint which is damaged due to prolonged contractures, trauma, or secondary to hand deformities after nerve damage. K-wire fixation is economical and simple method, that’s why it is used frequently in emergency settings. Pin tract infection is a common complication of K-wire fixation and in busy units like ours, where the turnover of the patients is very high, this risk of infection is relatively high. In our study, the older patients (>60 years of age) were excluded from the study because the chances of infection in this population is inherently higher and most of the patients in this age group also have co-morbidities like diabetes mellitus, ischemic heart disease, kidney, or lungs problems which also increase the risk of infection. Most of our patients who incurred pin site infection had modified Oppenheim score of 2, where there was redness and tenderness at the site of pin insertion but there was no discharge. This initial localized infection was treated with local (fusidic acid cream) and oral antibiotics (co-amoxiclav, dosage according to body weight), there was no need for removal of the pins or skin debridement. In the exposed K-wire group, there were only two patients where there was a need to remove the K-wire when local and systemic antibiotics did not help to control the discharge and slough. In one patient, the soft tissue involvement was severe and all the K-wires were infected so they had to be removed along with surgical debridement of the skin. In the buried K-wire group, there was only one patient where the pin had to be removed.

The patient in whom there was discharge from the pin site or where there was skin slough, wound culture was advised. Out of four patients where pins were removed three patients show growth of *Staphylococcus aureus *and one Pseudomonas species. These patients were treated with culture-specific antibiotics.

There was no correlation of soft tissue injury associated with bone fractures. The patients with moderate to severe soft tissue damage and with multiple fractures were already excluded from the study and it was made sure that all the pins were inserted through the healthy skin. Out of total 21 patients with infection, no one had skin damage at the site of insertion of K-wire.

In our study, the patients who were treated with buried K-wires the wires were inserted percutaneously or with open reduction but the end of the pin was cut close to the bone and the worn end was covered with skin. In the cases where multiple attempts were made in insertion of the wires, the wire insertion site was closed with a suture. After the fractures were healed and conformed on x-ray, the pins were removed in operation theater under local anesthesia with a small incision on the skin covering the pin insertion site and the end of the wire was grasped with a nose plier and pulled out. The wound was treated with local antibiotic ointment (fusidic acid cream) and dressing. Though there was an extra step of removing the K-wire, there was a significant decrease in the rate of infection with buried K-wires and there was better patient comfort and compliance, as with the exposed K-wire, there was a risk of pin being trapped with clothing or any other object which sometimes led to the removal of the pin and we had to insert the K-wires again. Such patients were omitted from the study.

Stern et al. reported that 42% of the patients in his series treated by plate fixation for metacarpal and phalangeal fractures developed infectious complications [21]. The plate fixation is more sophisticated method of hands fracture fixation but 42% infection rate is much higher than in our study where it is only 11.5% with buried K-wires with satisfactory outcomes and patient satisfaction and lower operation cost. Starker and Eaton also supported the use of K-wires for the fixation of hand fractures in emergency department due to its low complication rate as compared to screws and plate fixation in ER [22].

The overall infection rate of 14.7% in our study is also supported by Kalantri et al. where the overall infection rate was 18% [23]. Koc et al. conducted a prospective randomized trial with 104 patients and found an 8.8% incidence of infection for buried K-wires and 10% incidence of infection for exposed K-wire which was not statistically significant as compared to our study where it is 11.5% and 23% incidence of infection in buried and exposed K-wire patients respectively [24]. Similarly, Hsu et al. reported a 7% infection rate (24 of 359 pins) for exposed K-wires and 4% (2 of 49 pins) for buried K-wires, which is not statistically significant. In a study, there was an increased infection rate in metacarpal fractures as compared to phalangeal fractures, but in our study, there was no such difference [25].

The results of our study were comparable to a retrospective study by Botte et al., according to which there were 10 cases of infection out of 137 patients, two of the patients developed osteomyelitis. It is possible that pin site infection may be caused by Biofilm which grows on and tracks down the Kirchner wire which has been left in situ for approximately four weeks [26].

Buried K-wire fracture fixation technique was initially used by Faraj and Davis [27]. The benefits of burying the Kirchner wires are less incidence of infection and lower risks of early removal of an infected wire which may have repercussions as far as the fixation of the fracture is concerned. This may be particularly important with certain types of hand surgery. The disadvantage of burying Kirchner wires is that, in the majority, they will need to be removed in a day surgery unit where resources may already be constrained because this has considerable cost implications [28].

The incidence of infection increases with the length of time for which the Kirchner wires are left in situ particularly if left percutaneously for more than four weeks. Clasper et al. showed infection of the medullary canal when external fixators pins were contaminated with *Staphylococcus aureus* [29]. In our study, there was not a single patient who developed medullary osteomyelitis.

The limitation of this study was a limited sample size. Moreover, patients with multiple fractures were not included in the study. Furthermore, the variables such as mode of injury, characteristics of fracture, number of Kirchner wires used for fracture fixation, and surgeon’s expertise might have influenced the consequences of our study.

## Conclusions

Infection in bone is a dreadful complication in fracture injury hence, every step should be taken to prevent it from occurring. We conducted this study on fractures of metacarpals and phalanges fixed with K-wires to compare the occurrence of pin site infection in those who had buried Kirchner wire with those who had exposed Kirchner wire. In our study, we observed that the rate of infection is low in buried Kirchner wires as compared to exposed Kirchner wires, though not statistically significant (p>0.05), for the fractures of metacarpals and phalangeal fractures of hand. Hence we recommend that the K-wires be buried beneath the skin, i.e., subcutaneously. a number of existing randomized control trials and case-controlled studies established that there is a lower incidence of infection in buried Kirchner wires for fracture fixation and we have determined that buried Kirchner wires is a safer approach.
